# Cell immortalization facilitates prelamin A clearance by increasing both cell proliferation and autophagic flux

**DOI:** 10.18632/aging.203943

**Published:** 2022-03-08

**Authors:** Carlos González-Blanco, Patricia Marqués, Jesús Burillo, Beatriz Jiménez, Gema García, Manuel Benito, Carlos Guillén

**Affiliations:** 1 Department of Biochemistry, Complutense University, Madrid, Spain; 2 Centro de Investigación Biomédica en Red (CIBER) de Diabetes y Enfermedades Metabólicas Asociadas (CIBERDEM), Madrid, Spain; 3 Mechanisms of Insulin Resistance (MOIR2), General Direction of Universities and Investigation (CCMM), Madrid, Spain

**Keywords:** aging, Zmpste24, autophagy, proliferation, immortalization

## Abstract

Hutchinson-Gilford Progeria Syndrome is an ultrarare disease which is characterized by an accelerated senescence phenotype with deleterious consequences to people suffering this pathology. The production of an abnormal protein derived from lamin A, called progerin, presents a farnesylated domain, which is not eliminated by the causal mutation of the disease, and accumulates in the interior of the nucleus, provoking a disruption of nuclear membrane, chromatin organization and an altered gene expression. The mutation in these patients occurs in a single nucleotide change, which creates a de novo splicing site, producing a shorter version of the protein. Apart from this mutation, an alteration in the metalloproteinase Zmpste24, involved in the maturation of lamin A, causing a similar alteration than in progeria. However, in this case, patients accumulate a protein, called prelamin A, which generates similar alterations in the nucleus than progerin. The reduction of prelamin A protein levels facilitates the recovery of the phenotype in different mice models of the disease, reducing the aging process. Different strategies have been studied for eliminating this toxic protein. Here, we report that immortalization of primary cells derived from the Zmpste24 KO mice, facilitates prelamin A degradation by different mechanisms, being essential, the enhancing proliferative capacity that the immortalized cells present. Then, these data suggest that using different treatments for increasing proliferative capacity of these cells, potentially could have a beneficial effect, facilitating prelamin A toxicity.

## INTRODUCTION

Hutchinson-Gilford progeria syndrome (HGPS) is a deleterious and extremely rare disease associated with a premature aging phenotype. This disease is caused by a single autosomal mutation within exon 11 in the *LMNA* gene (*LMNA* 1824C>T), encoding lamin A protein, involved in the maintenance of inner nuclear membrane structure. This mutation results in the appearance of a cryptic splice site and resulting in the generation of a shorter version of the protein and constitutively farnesylated, which is called progerin [1]. Lamin A is the mature version of a protein which is synthesized as a pre-protein, known as prelamin A. Furthermore, it was described that the lack of a metalloproteinase called Zmpste24, which is involved in the maturation of pre-lamin A protein, also results in the appearance of the phenotype observed in both mice models of the disease as well as in human patients [2, 3]. The presence of the farnesylated protein is associated with the alteration in the integrity of nuclear structure, generating the appearance of nuclear blebs. In this regard, the prevention or the inhibition of this process can improve nuclear structure in HGPS [4, 5]. Apart from alteration in nuclear structure, there is a disruption of chromatin structure and an increase in DNA damage [6]. It has been published that the expression of progerin and other lamin A mutants clearly inhibits cell proliferation, inducing a premature senescence phenotype. Very interestingly, either p53 inhibition or the expression of the catalytic subunit of telomerase (hTERT) was able to block the defects associated with the expression of the mutant protein, progerin [7]. In this regard, very recently, the introduction of hTERT mRNA was associated with the reversion of several markers associated with the disease, including an increase in telomere length and cell proliferation and a decrease in cellular senescence and the senescence-associated secretory phenotype (SASP) [8]. In addition, the large subunit of the replication factor C (RFC1) is cleaved in HGPS cells. RFC1 is essential for the correct loading of proliferating cell nuclear antigen (PCNA) and DNA polymerase function for DNA replication [9]. Furthermore, a new negative regulator of cell proliferation in Zmpste24-deficient cells has been identified, known as miR-365 [10].

As it has been previously mentioned, HGPS cause the accumulation of the progerin in the dividing cells altering nuclear architecture and the appearance of nuclear blebbing. This protein accumulates and aggregates in the nuclei of cells. Autophagy is a catabolic process and it is involved in the elimination of several cellular components such as aggregates and dysfunctional organelles [11]. In this regard, rapamycin, a macrolide antibiotic and an inhibitor of mTORC1 signaling pathway, and several rapalogs has been associated with a reduction in the aggregates by the improvement of progerin clearance by the induction of autophagy [12, 13]. Very interestingly, treatment of HGPS cells with MG132, a proteasome inhibitor, favors progerin clearance by the activation of autophagy, as a compensatory mechanism, indicating a balanced and integrated system between both degradative processes [14].

Here, we report that as a consequence of cell immortalization in Zmpste24-deficient cells, there is a potent activation of autophagic flux and proliferative capacity, associated with very low levels of prelamin A protein. When we blocked the cell cycle, by the reduction of serum from 10% to 0,1%, it was revealed a dramatic accumulation of prelamin A. Our data suggest that immortalization of Zmpste24-deficient cells facilitates the elimination of toxic prelamin A by two mechanisms: induction of both proliferation and autophagy mechanisms.

## MATERIALS AND METHODS

### Antibodies and reagents

The following primary antibodies were obtained from Cell Signaling Technology (Beverly, MA, USA): anti-BiP (#3177), anti-LC3A/B (#4108), anti-Phospho-p70S6K (Thr389) (#9205), anti-p70S6K (#9202), anti-Phospho-ULK1 (Ser757) (#14202), anti-ULK1 (#6439), anti-phospho-PERK (Thr980) (#3179), anti-PERK (#3192), anti-Phospho-eIF2-alpha (Ser51) (#9721), anti-eIF2-alpha (#9722) anti-Tuberin/TSC2 (#4308). Anti-β-actin and anti-α-tubulin were obtained from Sigma-Aldrich (St. Louis, MO, USA). Anti-p62 (GP62-C-WBC) was obtained from ProGen (Heidelberg, Germany). Anti-Lamin A/C/B1 (EPR4068) was obtained from Abcam. Anti-prelamin A (#MABT345) was obtained from Millipore. Anti-p27 Kip1/CDKN1B (sc-1641) was obtained from Santa Cruz Biotechnology. The following secondary antibodies HRP-conjugated were used: anti-Rabbit (NA934) and anti-Mouse (NA931) were obtained from GE Lifesciences (Marlborough, MA, USA). Anti-Guinea pig HRP-conjugated (90001) was purchased by ProGen (Heidelberg, Germany). Chloroquine (C6628) was obtained from Sigma-Aldrich (St. Louis, MO, USA).

### Cell lines

Immortalized mouse embryo fibroblasts (MEF) from Zmpste24 WT and KO cells were kindly provided by Carlos López-Otín (University of Oviedo, Spain). These cell lines were cultured in 10% FBS DMEM high glucose supplemented and 10 mM HEPES. Primary fibroblast cultures from Zmpste24 WT and KO mice were generously donated by Carlos López-Otín (University of Oviedo, Spain). These cell lines were cultured in 15% FBS DMEM High Glucose medium supplemented with 20 mM HEPES and 1% non-essential amino acids (NEAA). The immortalization process was performed by retroviral infection with large T antigen of SV40.

### Western blotting analysis

After the different treatments, cells were washed twice with PBS 1x and lysed for protein extraction according to standard procedures. Alternatively, for determining the effect of the extraction method on the capacity to detect prelamin A in Zmpste24 KO cells, we proceed to sonication before protein concentration determination. Protein concentration was achieved by the Bradford method, using de Bio-Rad^®^ (Hercules, CA, USA) reagent and BSA as standard. Equal amounts of protein (15–20 μg) were submitted to electrophoresis and after SDS-PAGE gels were transferred to Immobilon P PVDF membranes (Merck Millipore, Burlington, MA, USA). Then, membranes were blocked with 5% BSA and incubated overnight with primary antibodies at 4ºC. The corresponding bands were visualized using the ECL Western blotting protocol (GE Healthcare, Little Chalfont, UK). Alternatively, for studying the role of cell cycle in prelamin A elimination, cells were cultured under normal growing conditions until reaching 70–80% of confluency. After that, the medium was discarded and we added a new medium with all the components apart from serum, which was added at 0,1% for 24 h. Then, cells were lysed, and the protocol was the same as previously described.

### Electron microscopy

Cell preparation for electron microscopy followed the protocol previously in [15]. Basically, cell pellets were fixed in a mixture of 4% paraformaldehyde (Electron Microscopy Sciences, 15710), 2,5% glutaraldehyde grade I (Sigma, G8882) in 0.1 M sodium phosphate buffer (pH 7.3) for 3 h. Then, the samples were postfixed in 1% OsO4 (Electron Microscopy Sciences, 19172) 1,5% K4[Fe(CN)6] during 1 h, dehydrated with ethanol and embedded in Epon812 (TAAB, T004). Thin sections (60–70 nm) were obtained with an Ultracut E (Leica) ultramicrotome, stained with lead citrate and examined under a JEM-1010 transmission electron microscope (JEOL).

### Immunofluorescence

Cells were grown on glass coverslips and fixed using paraformaldehyde 4% solution during 15–20 min., permeabilized in PBS with 0,5% Triton X-100 for 15 min. Then, the samples were blocked with blockage solution (3% BSA, 0,1% Tween 20 in PBS) for 1 hour. After that, cells were incubated overnight at 4ºC with primary antibodies (1:50 in blocking solution). After the incubation, coverslips were incubated with the corresponding secondary antibodies at a dilution of 1:100 for 1 hour 30 min. For colocalization analysis, the images were processed with Coloc2 (http://fiji.sc/Fiji). We chose different regions of interest (ROI) (at least 3 ROI of 3 different images) and the threshold was obtained automatically using Coste’s automatic threshold, determining the Pearson’s Correlation Coefficient (PCC).

### Statistical analysis

Statistically significant differences between mean values were determined using the unpaired Student *t*-test in the Graphpad statistical analysis software package. Alternatively, and when we compared several groups, we performed an ANOVA study using the Dunnett’s multiple comparison analysis as post hoc. Differences were considered statistically significant at *P* < 0.05, ^*^P < 0.05, ^**^P < 0.01, ^***^P < 0.001.

## RESULTS

### Immortalized Zmpste24 KO cells presents an increased in the basal autophagic flux

**Figure 1:**
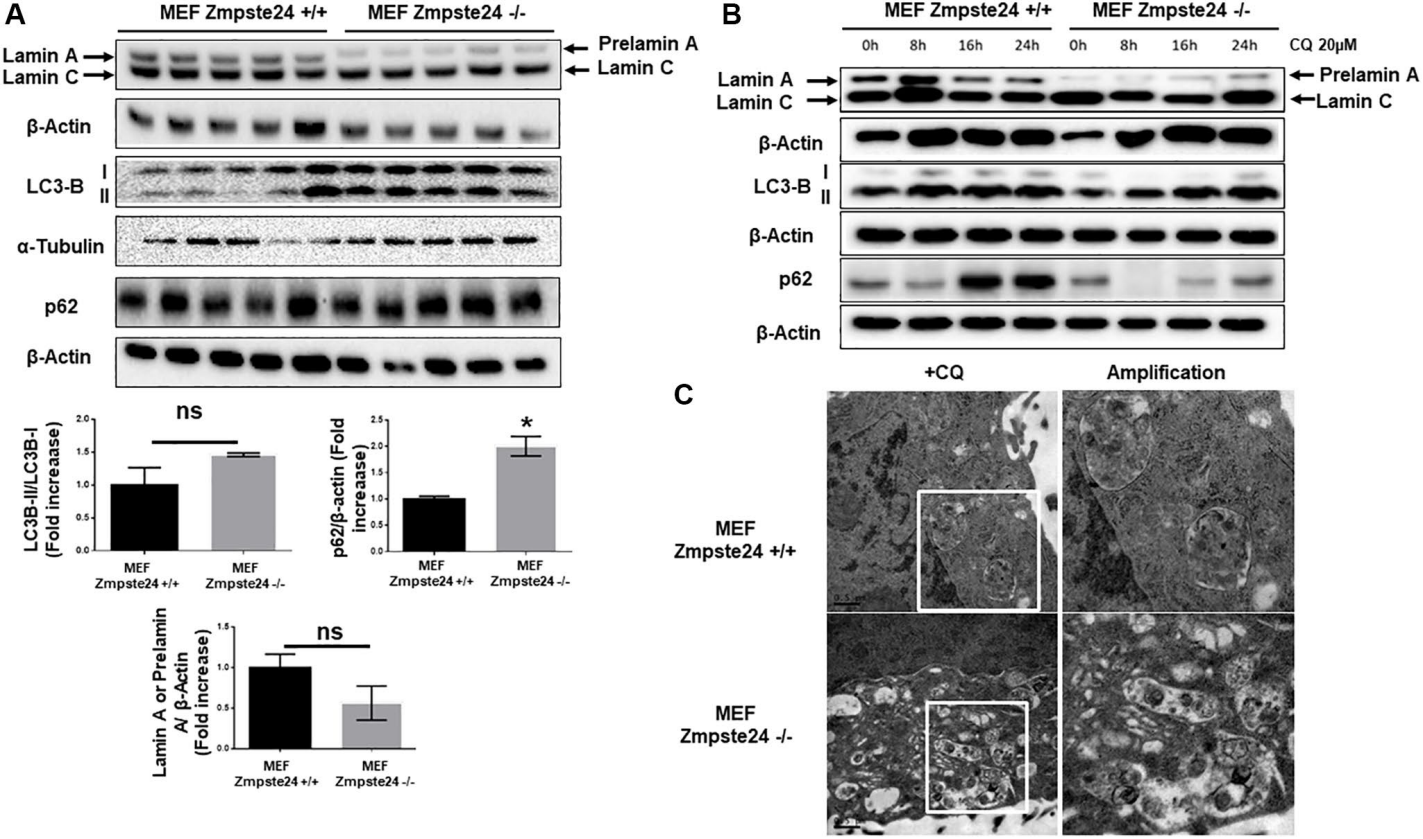
**Immortalized MEF Zmpste24 KO cells present an increase in the basal autophagic flux.** (**A**) Immunoblot analysis of Lamin A (in MEF Zmpste24 WT cells), prelamin A (in MEF Zmpste24 KO cells), LC3B II-I ratio and p62 using β actin as loading control, in the cell extracts in basal state (*n* = 5). The plot indicates the quantification data of Lamin A or prelamin A/β-actin ratio, p62/β-actin ratio and LC3B II/LC3B I ratio in the basal state. Data represent the mean ± standard error of the mean (SEM). Differences were determined by unpaired Student *t*-test analysis. ^*^p < 0,05 (*n* = 5). (**B**) Immunoblot analysis of Lamin A (in MEF Zmpste24 WT cells), prelamin A (in MEF Zmpste24 KO cells), LC3B II-I ratio and p62 using β actin as loading control, in the cell extracts treated with chloroquine (CQ) (20 μM) during 0, 8, 16 and 24 hours (*n* = 5). (**C**) Electron microscopy of MEF Zmpste24 WT and KO cells treated with CQ (20 μM) 24 hours. Enlargement of areas with accumulation of autophagosomes.

It is known that progeria phenotype is associated with an accumulation of senescent cells and a decreased proliferative capacity. Furthermore, there is an increase in the farnesylated form of prelamin A, which is toxic for cells. Firstly, we compared basal levels of different autophagic proteins in mouse embryo fibroblasts (MEF) with or without Zmpste24, generously donated by Carlos Lopez-Otín’s lab. First, we observed a statistically significant accumulation of LC3B-II protein levels with a concomitant accumulation in the adaptor protein p62 (Figure 1A). Unexpectedly, we observed a very low amount of pre-lamin A protein when we analyzed it by western blot analysis (Figure 1A). As prelamin A possesses a high hydrophobicity by its farnesyl groups, we obtained cell extracts by sonication, in order to increase protein solubility in our extracts. Using this approach, we did not observe any further accumulation of prelamin A protein compared with the cell extract obtained after this procedure (Supplementary Figure 1). In order to analyze basal autophagic flux, we pre-treated these cells with a low dose of CQ (20 μM), in order to not affect cell viability. CQ is an inhibitor of autophagic flux affecting to the autophagosome-lysosome fusion, by increasing lysosomal pH. After the addition of CQ, we observed an increased in LC3B-II/I ratio only in KO cells, suggesting that in Zmpste24-deficient cells, even at this low dose of CQ, there is high autophagic flux (Figure 1B and Supplementary Figure 2). Paradoxically, when we treated the cells with CQ, we did not observe an accumulation of prelamin A protein, which suggest the existence of alternative mechanisms, apart from autophagy, involved in prelamin A clearance and the low amount of this protein in these circumstances (Figure 1B). In addition, either under control conditions or after CQ treatment, a high colocalization signal between prelamin A and LC3B was also observed, indicating that autophagy is also involved in prelamin A degradation (Supplementary Figure 3). After electron microscopy analysis, we observed a massive accumulation of autophagosomes in response to CQ treatment in Zmpste24-deficient cells, corroborating a higher autophagic flux compared with WT cells (Figure 1C). In this regard, when we analyzed basal level of mTORC1 signaling, as one of the main negative regulators of autophagy, we clearly observed a reduction in the phosphorylation levels of both p70S6K (Thr 389) and ULK1 (Ser 757) with an associated increase in TSC2 protein levels (Supplementary Figure 4).

### Increased ER-stress in immortalized Zmpste24 KO cells

**Figure 2:**
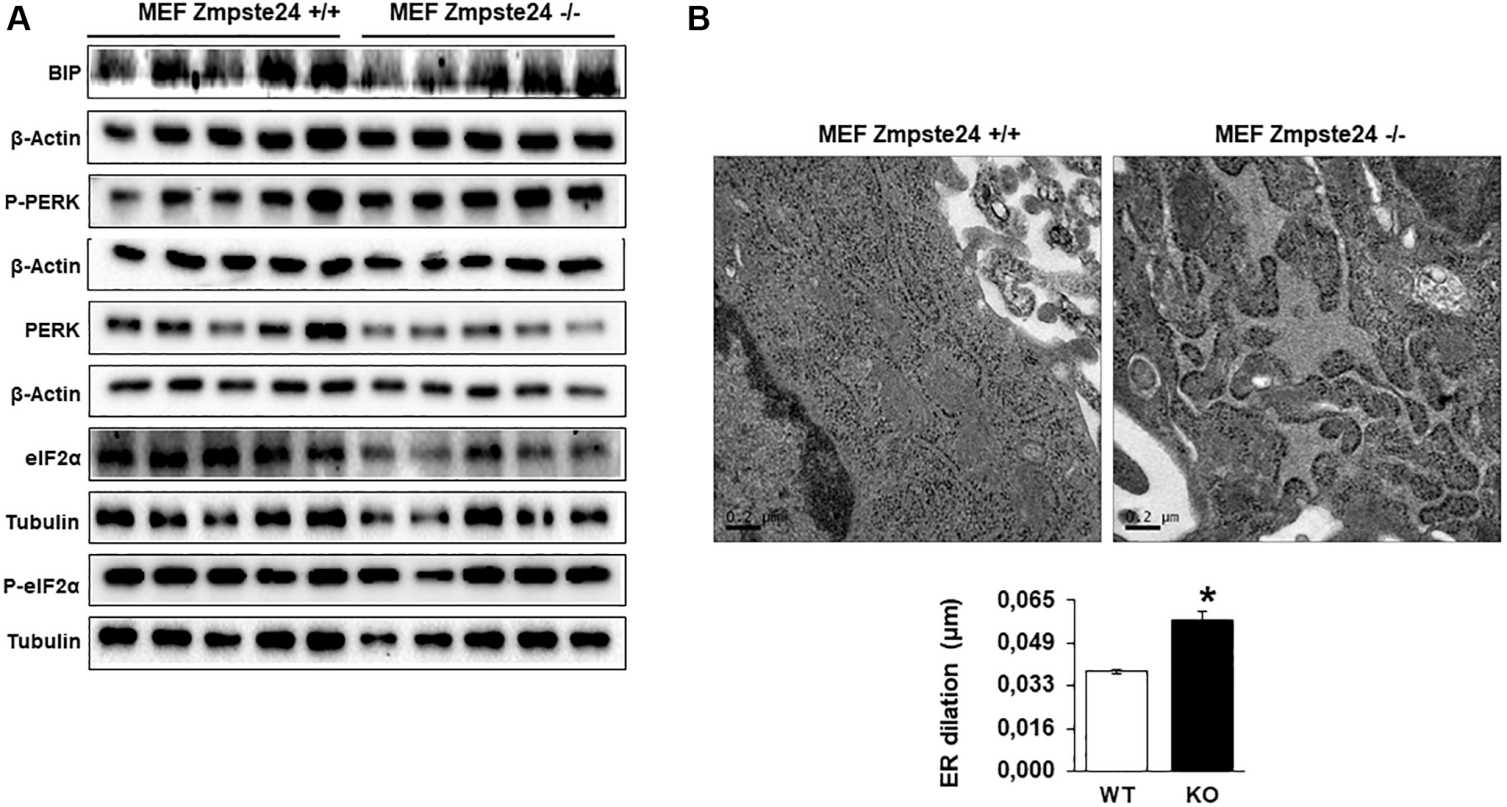
**Immortalized MEF Zmpste24 KO cells present an increase in ER-stress.** (**A**) Immunoblot analysis of BIP, P-PERK, PERK, eiF2α and P- eiF2α using both, β actin and Tubulin as loading control, in the cell extracts in basal state (*n* = 5). The plot indicates the quantification data of eiF2α/β-actin ratio, P-eiF2α 62/eiF2α ratio, BIP/Tubulin ratio and P-PERK/PERK ratio in the basal state. Data represent the mean ± standard error of the mean (SEM). Differences were determined by unpaired Student *t*-test analysis. ^*^p < 0,05; ^**^p < 0,01; ^***^p < 0,005 (*n* = 5). (**B**) Electron microscopy of ER of MEF Zmpste24 WT and KO cells in basal state. The plot indicates the quantification data of ER dilation using image J. ^*^p < 0,05.

Recently, it has been described that progerin induces ER-stress in vascular smooth muscle cells, facilitating either atherosclerosis or muscle dysfunction [16, 17]. Then, we decided to analyze ER-stress in our cells. An increase in the resident chaperone protein Bip/Grp78 was observed in Zmpste24 deficient cells compared with Zmpste24 wt cells (Figure 2A and Supplementary Figure 5). Very interestingly, we determined a reduction in the total amount of eIF2-α protein levels with a concomitant increased in both phospho-eIF2-α (Ser 51)/eIF2-α ratio and phospho-PERK (Thr 980)/PERK ratio in Zmpste24 KO cells (Figure 2A and Supplementary Figure 5). These data suggest that there is an activation of ER-stress in Zmpste24 deficient cells. To corroborate these data, a statistically significant increase in ER dilation in KO cells was also observed, providing another evidence of a basal activation of ER-stress in these cells (Figure 2B).

### Cell cycle contributes to the elimination of pre-lamin A in immortalized cells

**Figure 3:**
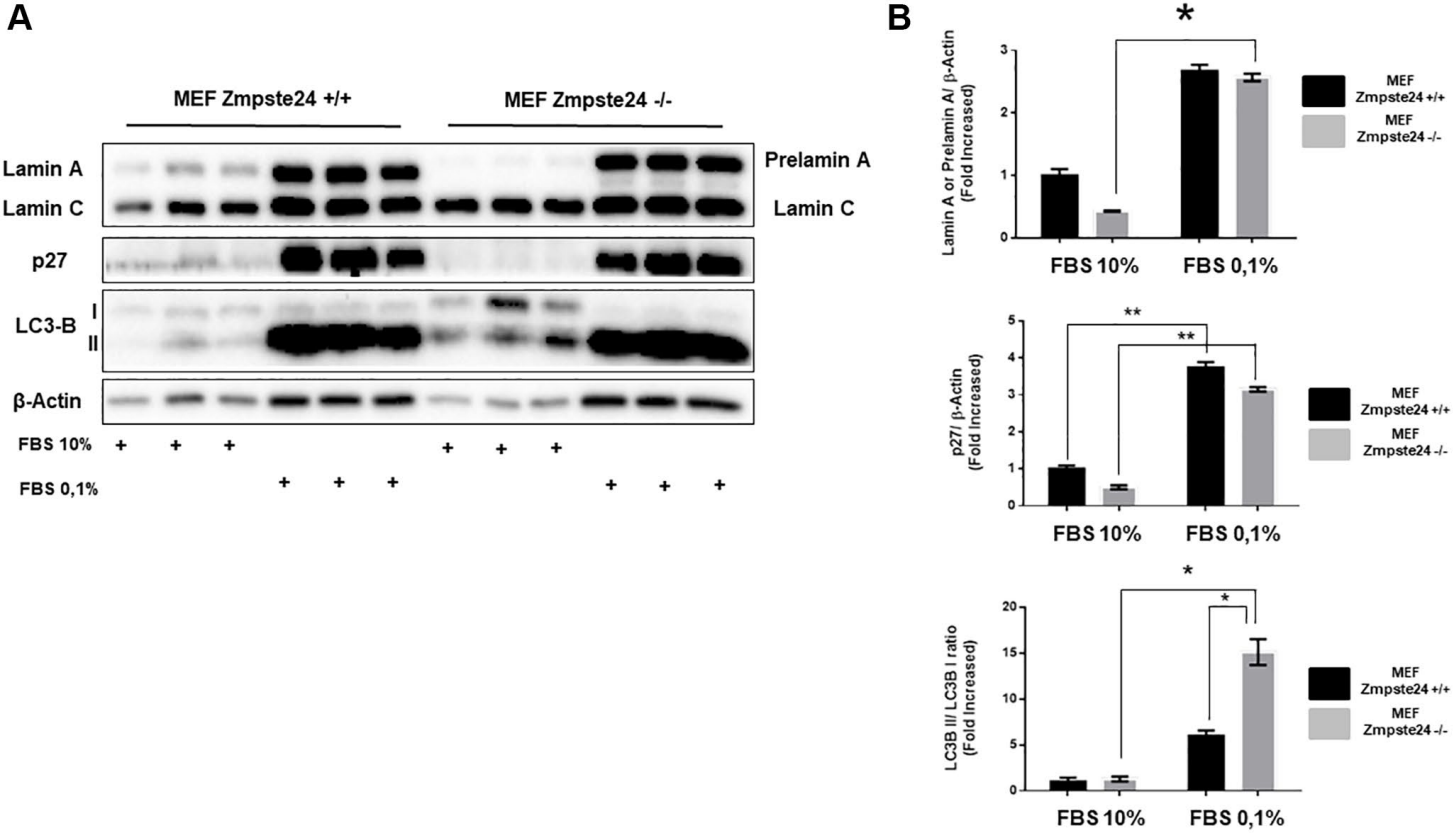
**The blockade of the cell cycle causes a prelamin A accumulation in immortalized MEF Zmpste24 KO cells.** (**A**) Immunoblot analysis of Lamin A (in MEF Zmpste24 WT cells), prelamin A (in MEF Zmpste24 KO cells), p27 and LC3B II-I ratio using β actin as loading control, in the cell extracts with FBS reduction (*n* = 3). (**B**) The plot indicates the quantification data of Lamin A or prelamin A/β-actin ratio, p27/β-actin ratio and LC3B II/LC3B I ratio of MEF Zmpste24 WT and KO in both in growth state and in nutrient deprivation. Data represent the mean ± standard error of the mean (SEM). Differences were determined by Dunnett's multiple comparisons test. ^*^p < 0,05; ^**^p < 0,01 (*n* = 3).

As previously shown, autophagy is involved in the elimination of pre-lamin A. However, the observation that using CQ we did not observe an accumulation of the protein, we decided to study alternative pathways for its elimination. Since the immortalized cells present an overactivation of the cell cycle and, it is known that progeria patients do not develop tumors despite high levels of DNA damage [18], we performed an experiment in which we inhibited cell cycle progression by the reduction of serum in the growth medium, for analyzing the consequences on prelamin A protein levels. Using this strategy, we clearly observed an accumulation of the suppressor protein p27, as expected, as well as an increase in the lipidated form of LC3B, in both cell lines (Figure 3A, 3B). Very importantly, after the reduction of serum for cell cycle arrest, we observed a huge accumulation of prelamin A protein. Furthermore, we observed an increase in LC3B-II protein levels after serum depletion in both cell lines (Figure 3A, 3B). Altogether, these data strongly suggest that cell proliferation capacity has a key role in prelamin A clearance.

### Primary cultures from Zmpste24 KO cells exhibit an increase in autophagic flux

**Figure 4:**
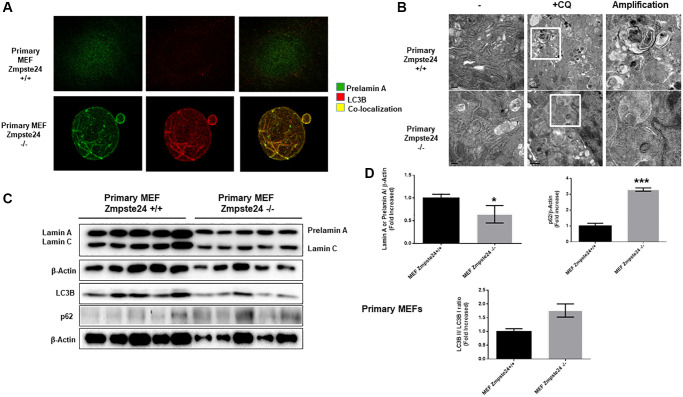
**Primary cells from Zmpste24 KO cells also exhibit an increase in autophagic flux.** (**A**) Immunofluorescence of Primary MEF Zmpste24 WT and KO cells under basal state using both an LC3B II/I antibody and a prelamin A antibody to see the co-localization signal. (**B**) Electron microscopy of Primary MEF Zmpste24 WT and KO cells in both basal states and treated with CQ (20 μM) during 24 hours. Enlargement of areas with accumulation of autophagosomes after treated with CQ. (**C**) Immunoblot analysis of Lamin A (in Primary MEF Zmpste24 WT cells), prelamin A (in Primary MEF Zmpste24 KO cells), LC3B II-I ratio and p62 using β actin as loading control, in the cell extracts in basal state (*n* = 5). (**D**) The plot indicates the quantification data of Lamin A or prelamin A/β-actin ratio, p62/β-actin ratio and LC3B II/LC3B I ratio in the basal state. Data represent the mean ± standard error of the mean (SEM). Differences were determined by unpaired Student *t*-test analysis. ^*^p < 0,05; ^***^p < 0,005 (*n* = 5).

In order to compare the effect of autophagy in primary cell cultures with immortalized cells, firstly, we determined that under basal state, Zmpste24 deficient cells presents an intense colocalization between prelamin A protein with the autophagic protein LC3B (average PCC in primary Zmpste24 WT cells 0,2+/−0,1 versus 0,9+/−0,1 in Zmpste24 KO cells), suggesting that autophagy could be involved in the degradation of pre-lamin A protein under control conditions in Zmpste24 deficient primary cells (Figure 4A). Then, when we pre-treated the cells with CQ, we observed an accumulation of autophagosomes in both Zmpste24 WT and KO cells by electron microscopy, pointing to an active autophagic flux, in a similar manner that we observed in immortalized cells (Figure 4B). In contrast, we did not observe any accumulation of prelamin A protein amount in Zmpste24 KO cells, suggesting that, although there is an active autophagic process, there is a failure in the clearance of prelamin A (Supplementary Figure 6), and suggested by the accumulation of both p62 and LC3B protein levels detected in these cells (Figure 4C, 4D). In addition, we observed an increase in the level of phospho-ULK1 (Ser 757) and a reduction in TSC2 (Supplementary Figure 7), although not statistically significant.

### ER stress is not increased in primary Zmpste24-deficient cells

**Figure 5:**
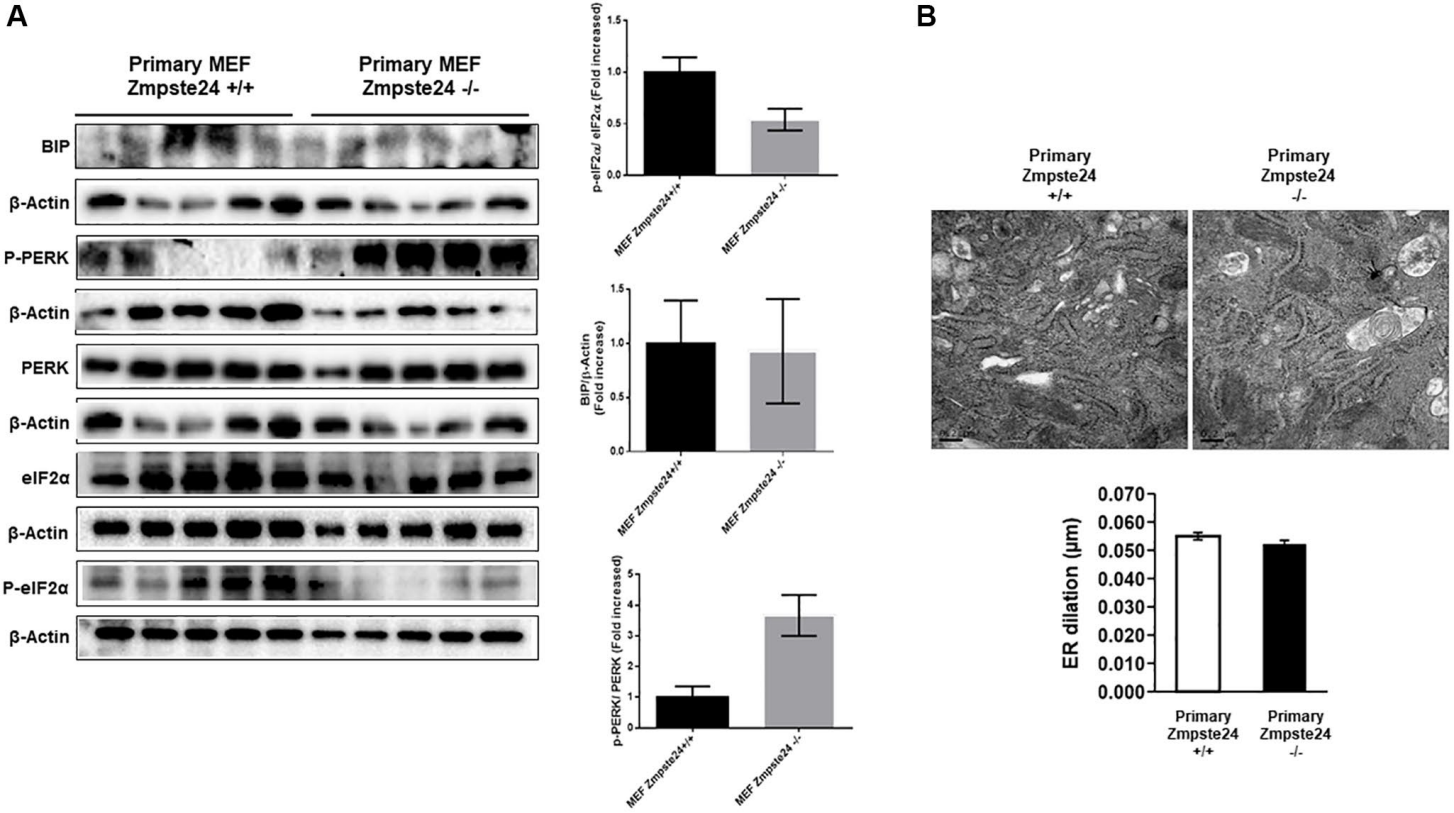
**ER stress is not increased in primary Zmpste24 KO cells.** (**A**) Immunoblot analysis of BIP, P-PERK, PERK, eiF2α and P- eiF2α using β actin as loading control, in the cell extracts in basal state (*n* = 5). The plot indicates the quantification data of eiF2α/β-actin ratio, P-eiF2α 62/eiF2α ratio, BIP/Tubulin ratio and P-PERK/PERK ratio in the basal state. Data represent the mean ± standard error of the mean (SEM). Differences were determined by unpaired Student *t*-test analysis. (**B**) Electron microscopy of ER of Primary MEF Zmpste24 WT and KO cells in basal state. The plot indicates the quantification data of ER dilation using Image J.

When we analyzed primary cells obtained from Zmpste24 WT and KO cells we observed no changes neither in the level of phospho-PERK (Thr 980)/PERK ratio nor phospho-eIF2-α (Ser 51)/eIF2-α ratio or Bip protein levels comparing both primary cells (Figure 5A). In this regard, both primary Zmpste24 WT and deficient cells presented an ER dilation being not statistically significant between both cell types (Figure 5B).

### Accumulation of pre-lamin a after cell cycle arrest

**Figure 6:**
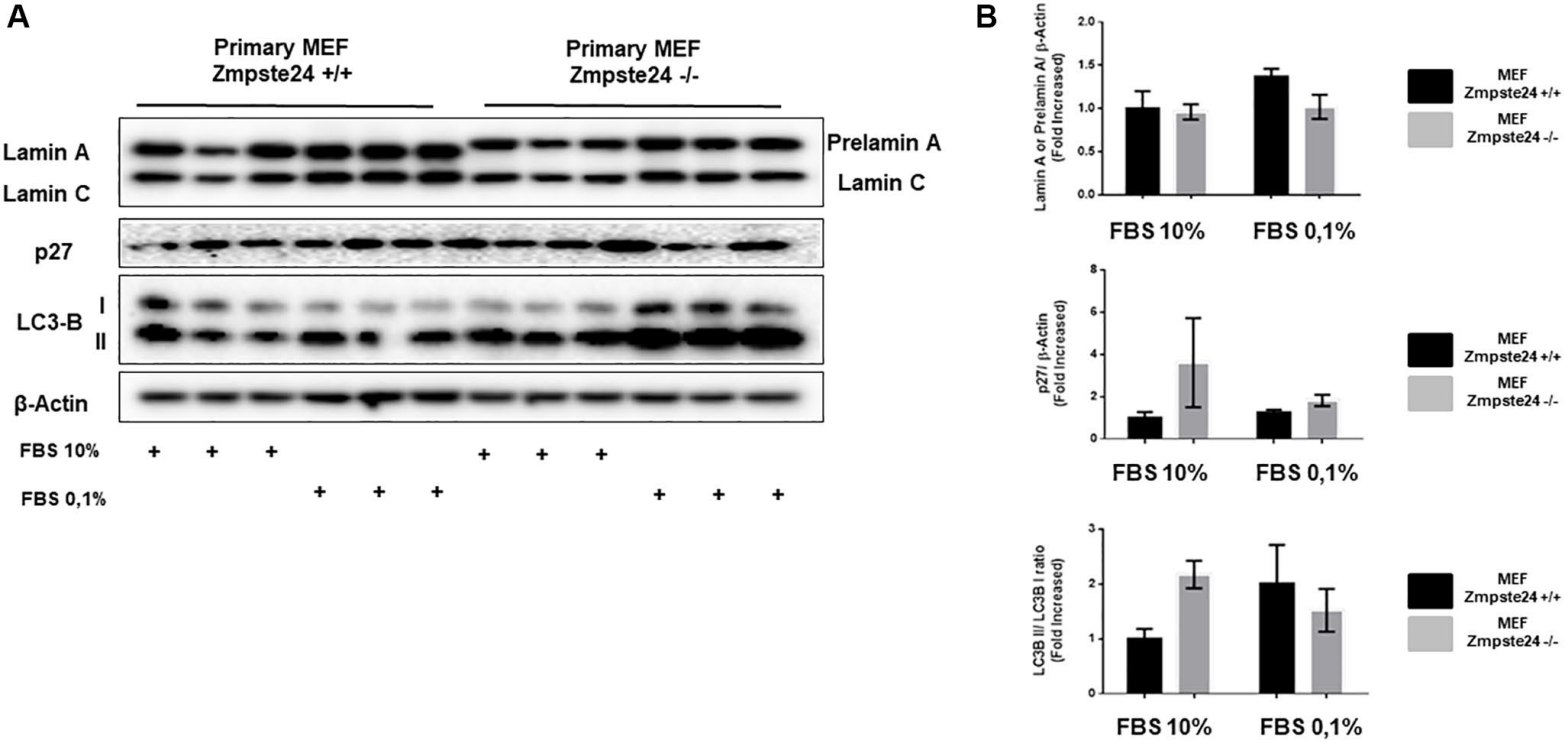
**Primary MEF Zmpste24 KO cells exhibit a blockade of the cell cycle in the basal state.** (**A**) Immunoblot analysis of Lamin A (in Primary MEF Zmpste24 WT cells), prelamin A (in Primary MEF Zmpste24 KO cells), p27 and LC3B II–I ratio using β actin as loading control, in the cell extracts with FBS reduction (*n* = 3). (**B**) The plot indicates the quantification data of Lamin A or prelamin A/β-actin ratio, p27/β-actin ratio and LC3B II/LC3B I ratio of Primary MEF Zmpste24 WT and KO in both in growth state and in nutrient deprivation. Data represent the mean ± standard error of the mean (SEM). Differences were determined by Dunnett's multiple comparisons test.

When we analyzed the effect of cell cycle arrest in primary cultures, we observed an important amount of prelamin A protein using normal growing conditions in Zmpste24 deficient cells. Interestingly, and differentially what we observed in the immortalized Zmpste24 deficient cells, no further increase in prelamin A protein levels was observed after arresting the cell cycle, by a reduction in serum concentration (Figure 6A). In addition, we detected an increase at the basal state of both p27 protein levels, as well as in LC3B-II/LC3B-I ratio in Zmpste24 KO cells. These data indicate the pro-senescent status of these cells and suggest a blockage in cell cycle progression and an increase in autophagosome formation, respectively (Figure 6B).

## DISCUSSION

Progeria is a devastating disease. Median life expectancy of people suffering this pathology is around 14–15 years and although all the tissues are affected, progeric patients die predominantly from cardiovascular complications. Classical mutations in HGPS lead to the accumulation of a toxic form of lamin A, called progerin, which is a farnesylated form of the protein. Then, the use of different farnesyltransferase inhibitors (FTIs) can reduce its toxicity [19]. There are multiple lines of research for treating people suffering this disease and very recently it has been approved by US food and drug administration (FDA) to administer in patients from one year of age and above with lonafarnib, which is a FTI [20]. However, there is no cure for this disease nowadays.

The alteration of mTOR signaling is related with aging and different age-related diseases including progeroid laminopathies [21]. In this regard, the use of rapamycin and some rapalogs such as everolimus and temsirolimus, are able to abolish some of the cellular alterations observed in HGPS patients such as nuclear blebbing, senescence and progerin accumulation [12, 13, 22]. mTOR is a serine/threonine kinase involved in the control of main processes inside the cells, such as proliferation and autophagy [23]. Then, the modulation of this pathway has profound consequences on cell viability and survival. In this paper, we propose that in Zmpste24 deficient cells, as a consequence of the *in vitro* immortalization, there is an increase in both processes, cell proliferation and autophagic flux. When we compared primary and immortalized Zmpste24 KO cells, it is evident that in the immortalized Zmpste24 KO cells there is a significant reduction in mTORC1 signaling with a concomitant increase in TSC2 protein levels, which is coherent with the activation of autophagic flux that we observed in these cells. However, in primary Zmpste24 KO cells, although the changes were not significant, there is a tendency to see an activation of mTORC1 signaling and a reduction of TSC2, which is just the opposite scenario that we do see in the immortalized cells. In addition, it is in the immortalized Zmpste24 KO cells where we do not observe an accumulation of prelamin A, just the contrary that occurs in the primary cells. All of these data fit perfectly with results published very recently, indicating that accumulation of prelamin A facilitates the appearance of premature aging, through the hyperactivation of mTORC1 [24].

Autophagy activation is a degradation process which has been involved in prelamin A degradation [25]. As it was mentioned before, the use of rapamycin has beneficial effects in different aspects of the disease. Rapamycin, apart from being a mTOR inhibitor, is a potent autophagy inducer. In fact, rapamycin decreases the formation of insoluble progerin aggregates and facilitates its clearance through autophagy activation [12]. In addition, prelamin A clearance has been also associated with an activation of autophagy [26]. In this regard, the inhibition of proteasome activates autophagy and hence favours progerin degradation [14]. Our data indicate that in both cell types, immortalized and primary Zmpste24 KO cells, there is an increase in the basal autophagic flux. In this regard, it has been described that in a HGPS mice model, although it is known that autophagy decreases with age, there is an increase in the basal activation of autophagy [27]. However, when we blocked autophagy by a pretreatment of immortalized cells with CQ, we did not observe any accumulation of prelamin A, suggesting that this mechanism is not essential for protein degradation of prelamin A. Similar results were obtained with primary cells. Then, we can conclude that autophagy has a minor role in prelamin A degradation in immortalized cells, and alternative mechanisms are probably necessary for explaining the low prelamin A protein levels observed in Zmpste24 KO cells. Interestingly, lamin B1 has a higher binding capacity with LC3B than lamins A/C and B2, suggesting a lower degradative mechanism of lamins A/C and B2 by an LC3B-dependent mechanism under tumorigenic stimuli [28]. However, the binding capacity of prelamin A to LC3B has not been analyzed yet. Although our results indicate that macroautophagy is not essential in the elimination of prelamin A, we cannot rule out the involvement of other types of autophagy in prelamin A clearance, such as microautophagy or nucleophagy [29].

Prelamin A accumulation alters DNA replication because of the destabilization of the interaction between Lamin A and PCNA, by a competition mechanism, contributing to genomic instability and DNA replication fork stalling [30]. In fact, one of the key components for the correct function of DNA polymerases in DNA replication, a protein called RFC1, is degraded in HGPS patients [9]. When we analyzed the consequences of cell cycle arrest in immortalized cells, we evidenced several differential results in comparison with the primary cultures. First of all, we observed higher levels of prelamin A under normal growth conditions in primary Zmpste24 deficient cells than in the corresponding immortalized cells, suggesting that immortalized cells have a higher capacity to eliminate the toxic protein than primary cells. Furthermore, when we diminished cell cycle progression, by the reduction of serum percentage, we observed a huge accumulation of prelamin A only in immortalized Zmpste24 KO cells. These changes were not detected in the primary cells. Another important difference is the undetectable level of p27 protein levels in immortalized Zmpste24 WT and deficient cells compared to the corresponding primary cells under normal growing conditions. These changes are compatible with the senescent phenotype observed in primary cells, which is reverted after immortalization. One possible interpretation of all these data is that primary Zmpste24 KO cells presents a senescent phenotype, with a reduced proliferation capacity, and an accumulation of prelamin A, which could be involved in a mild activation of mTORC1 activation. However, and probably as a consequence of the immortalization process, immortalized Zmpste24 KO cells presented a basal decrease in p27 protein levels associated with an almost undetectable level of prelamin A protein, probably by an improved replication capacity of these cells and related with a higher autophagic flux. In addition, immortalized Zmpste24 KO cells presented a decrease in mTORC1 signaling pathway, probably by a reduction in the toxicity of prelamin A accumulation, as it has been recently proposed [24].

Senescence is a protective mechanism that blocks proliferation in cells limiting oncogenic transformation [31, 32]. Then, potentiating this capacity could have protective effects in progeroid cells. In this regard, very recently it has been published that the reintroduction of purified human telomerase mRNA can revert some of the abnormalities observed in HGPS cells. These changes included an increase in proliferative capacity and cellular lifespan [8]. In this regard, it has been uncovered that in Zmpste24 KO cells, there is a down-regulation in miR365, which is involved in cell proliferation. Then, the overexpression of this miRNA in Zmpste24 KO cells potentiates proliferation and a reduction in the number of senescent cells. However, its inhibition is linked to a decreased proliferative capacity and an accumulation in the number of senescent cells [10]. Similarly, our results indicate that immortalization generates a reactivation of cell proliferation and an increase in the autophagic flux, which potentiates prelamin A elimination. Very importantly, genetic reprogramming of progeria fibroblasts has been demonstrated to modify and reconstruct a normal epigenetic landscape, revitalizing cells to a pluripotent and more proliferative state, constituting a very interesting avenue of research for a future treatment of progeria disease [33]. However, total reprogramming has been linked to the appearance of different tumors in animal models, limiting its use in patients. However, it has been published a very interesting alternative, which is the partial reprogramming of the cells, in order to diminish the malignant transformation of cells, reducing deleterious effects of reprogramming [34]. In this regard, this strategy has been used *in vivo* demonstrating its effect in reversing aging with no effects in tumorigenesis [35].

**Figure 7:**
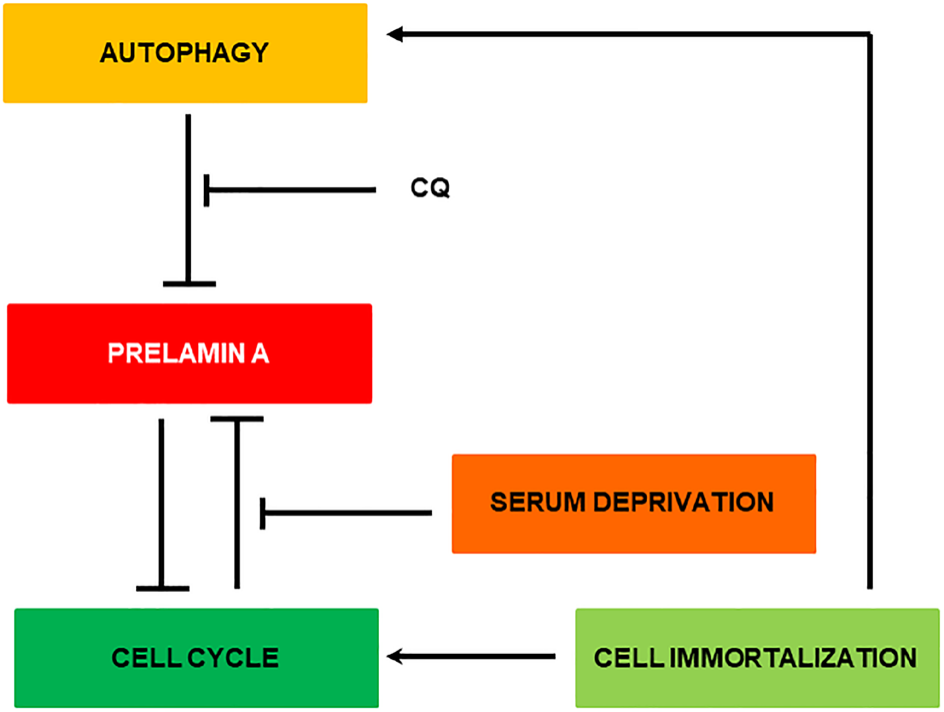
Representative scheme of the possible clearance mechanism of prelamin A in MEF Zmpste24 KO.

In Figure 7 is depicted the most relevant observations obtained in this manuscript. Basically, removal of Zmpste24 causes a natural accumulation of prelamin A anchored to the cell nucleus. Initially, this accumulation can be partially removed by autophagy. This process is much more likely to occur when the cell is dividing since for this the nuclear structure is broken and prelamin A bound to LC3B is more accessible to the cell’s degradative machinery. In fact, it is known that autophagy can degrade nuclear components in response to tumorigenesis [28]. When this state is prolonged in time, more prelamin A accumulates, causing DNA damage and inhibiting cell division, creating a positive feedback loop between the accumulation of prelamin A and the blockage of the cell cycle. When the cell is immortalized, the accumulation of prelamin A is diminished by the activation of cell cycle progression and autophagy processes. Then, that positive feedback loop is not generated and with each division the cell is able to reduce the levels of prelamin A to fewer levels allowing cell viability.

In conclusion, although autophagy modulation has been implicated in prelamin A elimination, our data indicate that in the immortalized cell lines, cell cycle progression has a major role in prelamin A clearance. Then, in this paper we propose that the reactivation of cell proliferation in Zmpste24 KO cells represents a very important strategy for diminishing prelamin A protein and facilitating an anti-aging effect in these cells.

## Supplementary Materials

Supplementary Figures
