# Supplementary material for: Cell immortalization facilitates prelamin A clearance by increasing both cell proliferation and autophagic flux
Source: Aging (Albany NY). 2022 Mar 8;14(5):2047–61. doi: 10.18632/aging.203943 (PMC8954962; doi:10.18632/aging.203943)
Supplement: Supplementary Figures [file aging-14-203943-s001.pdf]

## SUPPLEMENTARY FIGURES

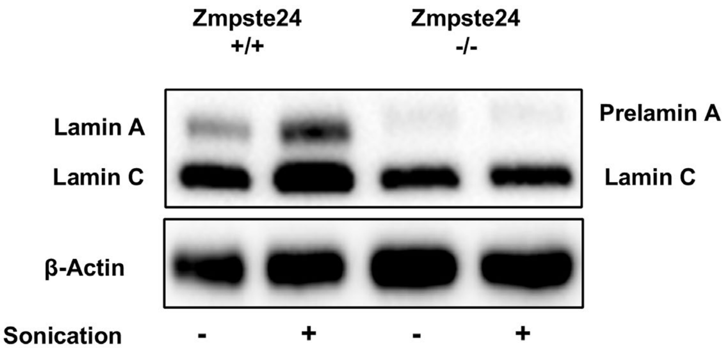


**Supplementary Figure 1. Immunoblot analysis of Lamin A (in MEF Zmpste24 WT cells) and prelamin A (in MEF Zmpste24 KO cells) after sonication of cell extracts, using β-actin as loading control (*n* = 1).**

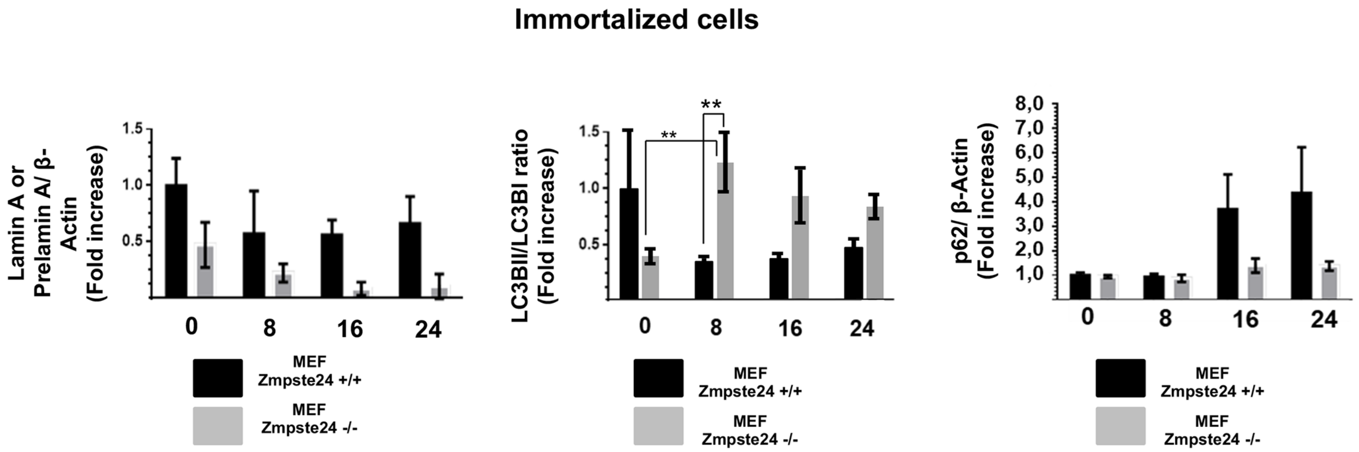


**Supplementary Figure 2. Quantification data of Lamin A or prelamin A/β-actin ratio, p62/β-actin ratio and LC3B II/LC3B I ratio in the cell extracts treated with CQ (20 µM) during 0, 8, 16 and 24 hours (*n* = 5).** Data represent the mean ± standard error of the mean (SEM). Differences were determined by Dunnett's multiple comparisons test. $^{**}p < 0{,}01$ (*n* = 5).

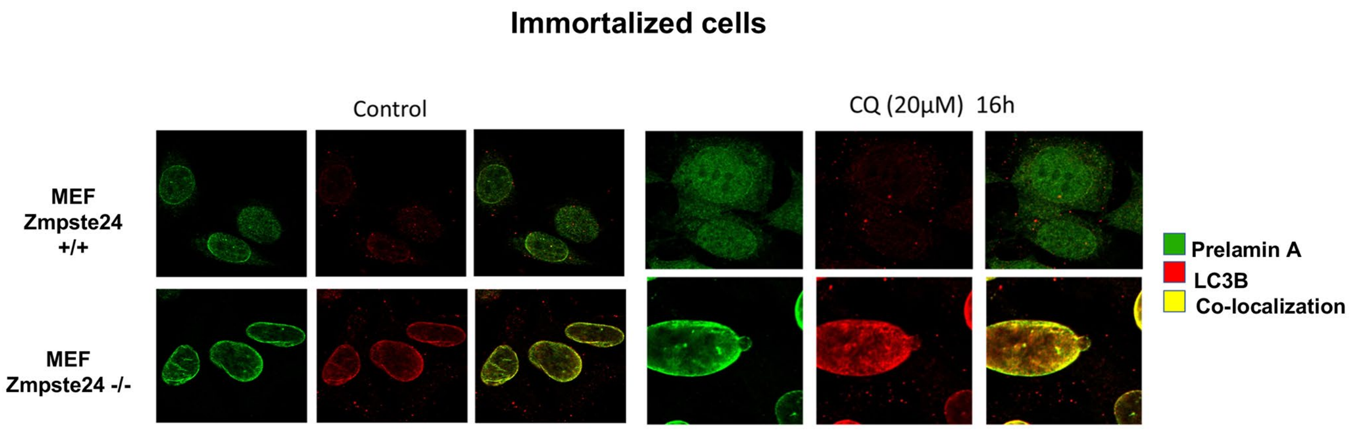


**Supplementary Figure 3. Immunofluorescence of MEF Zmpste24 WT and KO cells under basal state and treated with CQ (20 µM) during 16 hours using both an LC3B II/I antibody and a prelamin A antibody to see the co-localization signal.**

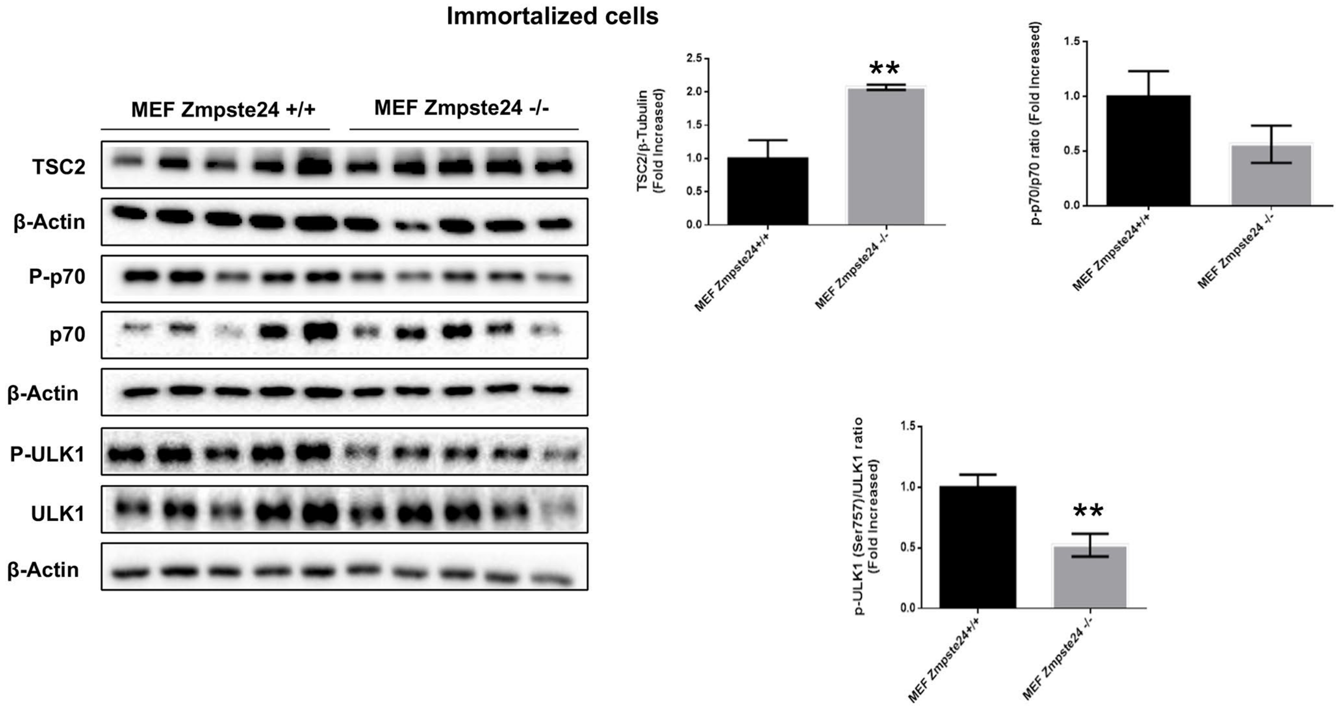


**Supplementary Figure 4. mTORC1 pathway**. Immunoblot analysis in immortalized MEF Zmpste24 WT and KO cells of TSC2, p-p70/p70 ratio, p-ULK-1/ULK ratio using β actin as loading control, in the cell extracts in basal state (*n* = 5). Quantification data of TSC2/β-actin, P-p70/p70 ratio, P-ULK-1/ULK ratio in basal state (*n* = 5). Data represent the mean ± standard error of the mean (SEM). Differences were determined by unpaired Student *t*-test analysis. $^{**}p < 0{,}01$ (*n* = 5).

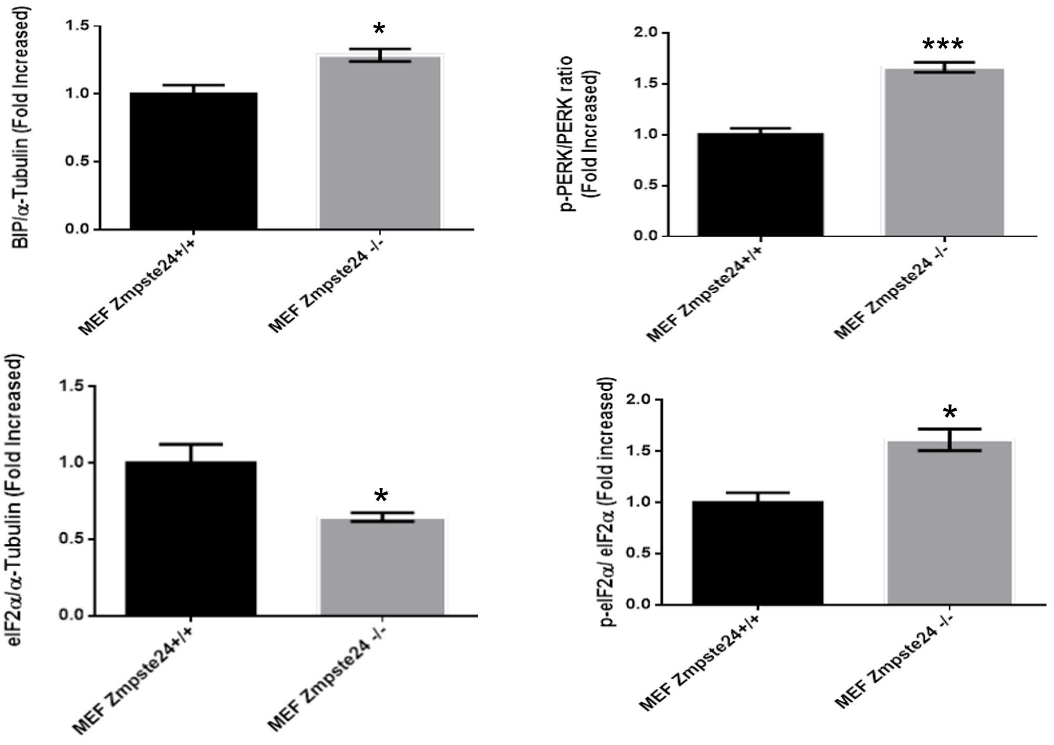


**Supplementary Figure 5. Quantification and statistical analysis of BIP/Tubulin; phospho-PERK/PERK; phospho-eIF2-alpha/eIF2-alpha and eIF2-alpha/Tubulin.** Data represent the mean ± standard error of the mean (SEM). Differences were determined by unpaired Student *t*-test analysis.

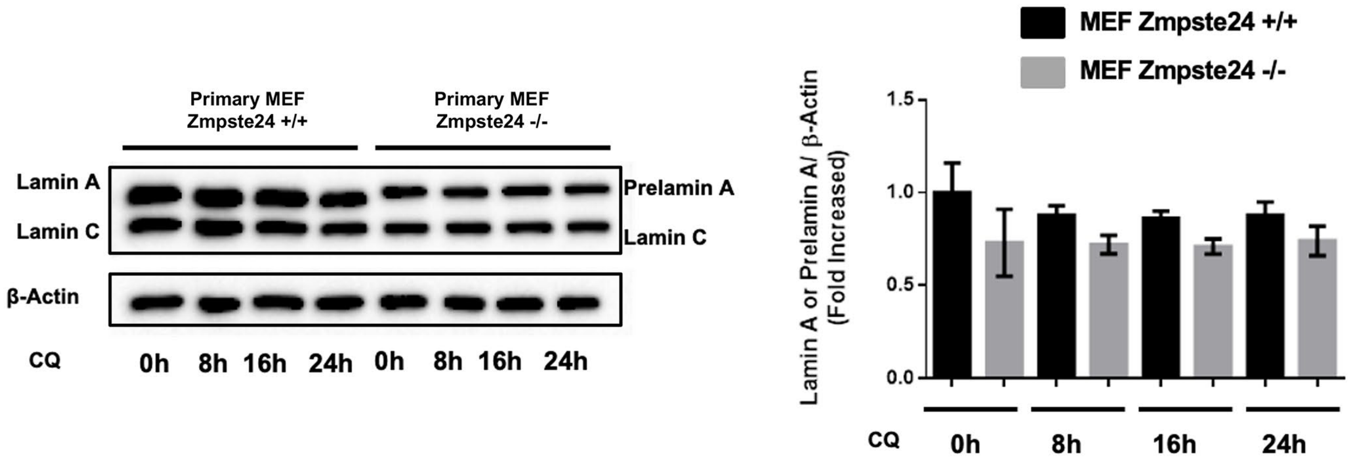


**Supplementary Figure 6. Immunoblot analysis of Lamin A (in Primary MEF Zmpste24 WT cells) and prelamin A (in Primary MEF Zmpste24 KO cells), using β actin as loading control, in the cell extracts treated with CQ (20 μM) during 0, 8, 16 and 24 hours (*n* = 5).** The plot indicates the quantification data of Lamin A or prelamin A/β-actin ratio in the cell extracts treated with CQ (20 μM) during 0, 8, 16 and 24 hours (*n* = 5). Data represent the mean ± standard error of the mean (SEM). Differences were determined by Dunnett's multiple comparisons test.

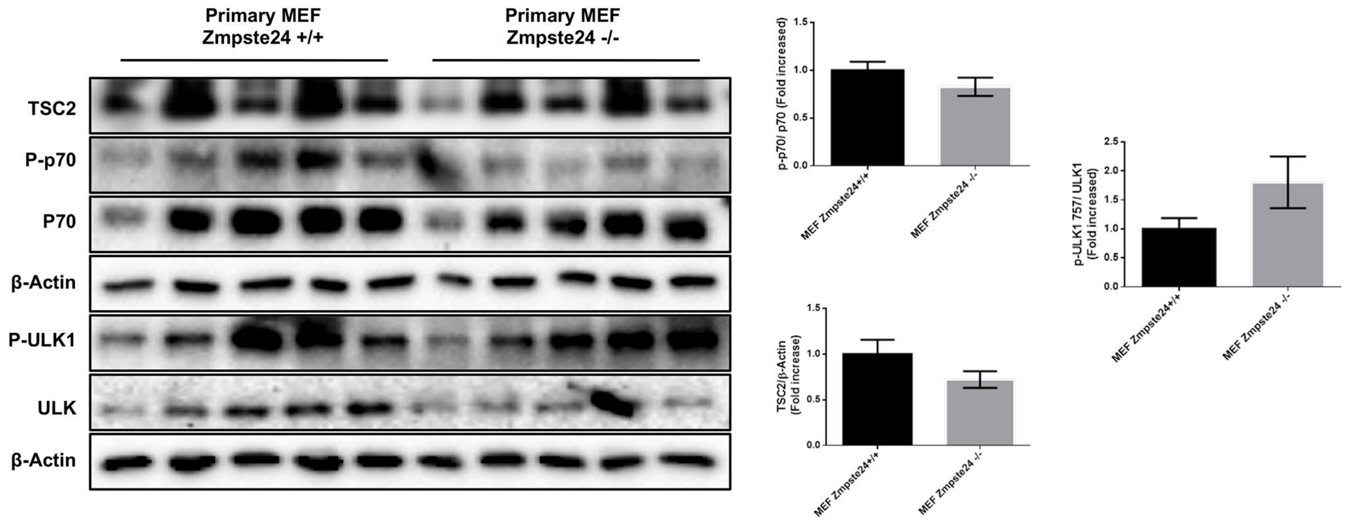


**Supplementary Figure 7. mTORC1 pathway.** Immunoblot analysis in Primary MEF Zmpste24 WT and KO cells of TSC2, p-p70/p70 ratio, p-ULK/ULK ratio using β-actin as loading control, in the cell extracts in basal state (*n* = 5). Quantification data of TSC2/β-actin, P-p70/p70 ratio, P-ULK/ULK ratio in basal state (*n* = 5). Data represent the mean ± standard error of the mean (SEM). Differences were determined by unpaired Student *t*-test analysis.
